# Acquired leukonychia with lichenoid papules

**DOI:** 10.1016/j.jdcr.2024.11.023

**Published:** 2024-11-30

**Authors:** Elizabeth Scheuble Mauk, Robert Dazé

**Affiliations:** a Marian University, Tom and Julie Wood College of Osteopathic Medicine, Indianapolis, Indiana; b Forefront Dermatology, Noblesville, Indiana

**Keywords:** amyopathic dermatomyositis, connective tissue disease, dermatomyositis, leukonychia

## Case

**Fig 1:**
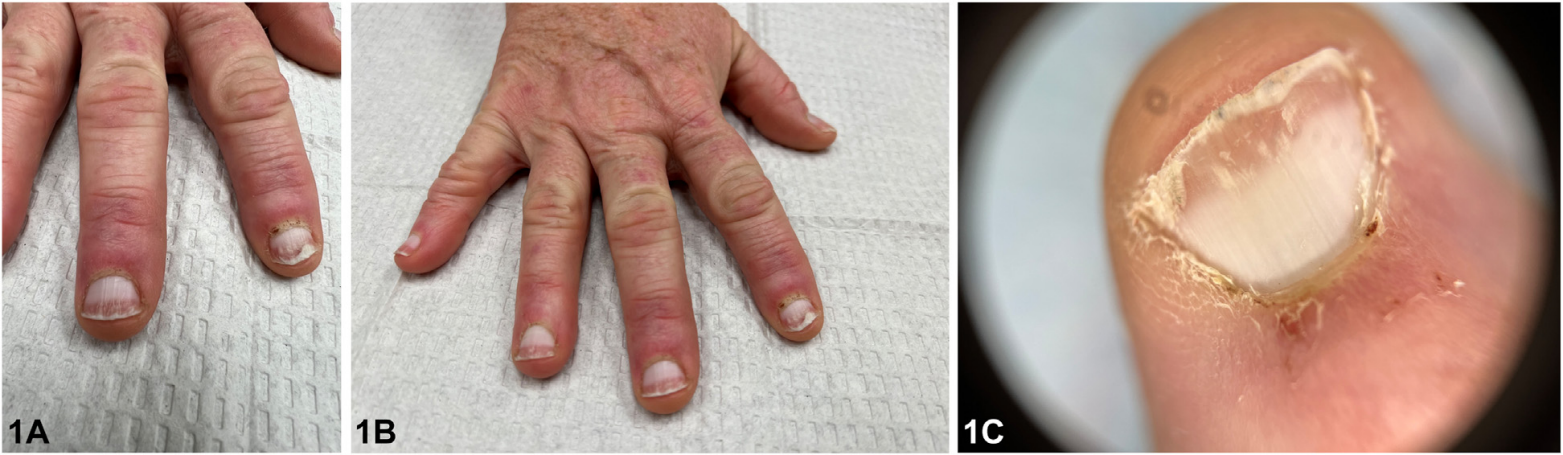

A 54-year-old female with a history of type 2 diabetes, renal insufficiency (estimated glomerular filtration rate >60 mL/min/1.73 m^2^) and basal cell carcinoma presented for a routine skin examination. Physical exam revealed scattered pink to violaceous papules on distal interphalangeal joints sparing the interphalangeal spaces, hyperkeratotic cuticles with punctate hemorrhages, onycholysis of the distal nail plates, and apparent leukonychia of the proximal nail plates (Fig 1, *A*-*C*). She denied other cutaneous involvement and the remainder of the skin was unremarkable. Muscle enzymes and antinuclear antibody were within normal limits. Labs were remarkable for elevated anti-isoleucyl-transfer RNA synthetase antibody and antitranscription intermediary factor 1 gamma (anti-TIF1-γ) antibody.

**Question 1: What is the most likely diagnosis?**

A. Amyopathic dermatomyositis (DM)
B. Systemic sclerosis (SSc)
C. Systemic lupus erythematosus (SLE)
D. Superficial white onychomycosis
E. Psoriasis

**Answers:**

A. Amyopathic dermatomyositis (DM) – Correct. DM is an inflammatory myopathy characterized by cutaneous, muscle, and systemic manifestations. Pathognomonic cutaneous involvement of DM includes Gottron papules overlying the metacarpophalangeal and the interphalangeal joints, as well as dystrophic cuticles with dilated capillaries and vessel drop out.^1^ The patient’s kidney function was noted to be well controlled and it remains unclear if the leukonychia was entirely secondary to her renal insufficiency or a novel association with DM.
B. Systemic sclerosis (SSc) – Incorrect. SSc is an autoimmune connective tissue disease characterized by cutaneous sclerosis that commonly presents in the fingers and hands.^2^ Abnormal nail-fold capillaries are present in the majority of SSc patients and appear very similar to those in DM, however pitting edema of the fingers is usually an early sign of SSc and an accompanying finding on examination.^2^ Furthermore, Gottron papules would not be present in SSc.
C. Systemic lupus erythematosus (SLE) – Incorrect. SLE is a multisystem disease that often has cutaneous manifestations and similar nail-fold and nail plate changes including transverse leukonychia.^2^^,^^3^ The patient’s clinical presentation, labs, and antibody profile including the negative antinuclear antibody exclude SLE as a diagnosis.
D. Superficial white onychomycosis – Incorrect. Superficial white onychomycosis is a dermatophyte infection of the nail plate, classically due to *T. interdigitale*, leading to discrete white patches that can be removed with a scalpel.^2^ This is in contrast to apparent leukonychia, which is due to nail bed edema and fades with pressure.^2^
E. Psoriasis – Incorrect. The characteristic nail plate changes associated with psoriasis include nail pitting, oil drop changes, and transverse leukonychia.^2^^,^^3^ There can be nail-fold inflammation with loss of the cuticles but without abnormal capillaries.^2^

**Question 2: With the patient’s anti-TIF1-γ antibody, she is at increased risk for which of the following?**

A. Vasculopathy
B. Malignancy
C. Necrotizing myopathy
D. Interstitial lung disease
E. Dysphagia

**Answers:**

A. Vasculopathy – Incorrect. Cutaneous vasculopathy can result in painful ulcerations seen in adult DM. This is clinically associated with the anti-melanoma differentiation-associated gene 5 (MDA5) antibody.^1^
B. Malignancy – Correct. The anti-TIF1γ antibody as well as the antinuclear matrix protein 2 antibody portend an increased risk of malignancy in adults, both solid organ and hematologic. There is no increased risk within the pediatric population. The malignancy rates range between 20% and 65%.^1^
C. Necrotizing myopathy – Incorrect. Antisignal recognition particle antibody is a non-DM-associated myositis-specific antibody and is associated with severe and sudden onset progressive muscle weakness.^1^ The patient’s current antibody profile does not increase the risk for necrotizing myopathy.
D. Interstitial lung disease – Incorrect. Pulmonary disease is a common systemic manifestation of adult DM affecting 15% to 30% of patients.^2^ It is most associated with the anti-MDA5 antibody and a notable feature of antisynthetase syndrome.^1^^,^^2^
E. Dysphagia – Incorrect. In adult DM, the gastrointestinal tract can be affected resulting in dysphagia and other motility issues. This is strongly associated with the antismall ubiquitin-like modifier activating enzyme antibody.^1^

**Question 3: What is the most appropriate first-line treatment for mild to moderate cutaneous disease?**

A. Methotrexate
B. Hydroxychloroquine
C. Dapsone
D. Rituximab
E. Apremilast

**Answers:**

A. Methotrexate – Correct. In addition to sun protection, topical corticosteroids or calcineurin inhibitors, methotrexate titrated up to 25 mg orally weekly is considered a first line therapy for mild to moderate cutaneous DM. If myopathy is present, this should be combined with systemic corticosteroids.^4^
B. Hydroxychloroquine – Incorrect. This antimalarial was previously considered a first line agent, but recent evidence has demonstrated that patients treated with this medication are likely to flare their cutaneous disease. Given the favorable safety profile, hydroxychloroquine should be considered as an adjuvant medication when the cutaneous disease is not controlled by other pharmacotherapies.^4^
C. Dapsone – Incorrect. Dapsone has been used for cutaneous DM, but the clinical evidence to support this medication as a first-line agent is lacking and not well elucidated in the literature.^2^
D. Rituximab – Incorrect. When systemic corticosteroids and a steroid-sparing immunosuppressive agent (ie methotrexate or mycophenolate mofetil) have failed, rituximab should be a considered an appropriate second line therapy. Rituximab with systemic corticosteroids would be considered first line if the patient presented with evidence of vasculopathy, calcinosis, or was positive for the anti-MDA5 antibody.^4^
E. Apremilast – Incorrect. While apremilast is an emerging treatment for DM and currently administered off-label, its therapeutic role in management of DM is not well defined as compared to other systemic therapies.^4^

## Conflicts of interest

None disclosed.
